# Supplementary material for: The Impact of Healthy Lifestyles on Late Sequelae in Classical Hodgkin Lymphoma and Diffuse Large B-Cell Lymphoma Survivors. A Systematic Review by the Fondazione Italiana Linfomi
Source: Cancers (Basel). 2021 Jun 23;13(13):3135. doi: 10.3390/cancers13133135 (PMC8268176; doi:10.3390/cancers13133135)
Supplement: Supplementary file 1 [file cancers-13-03135-s001.zip › cancers-1215681-supplementary/cancers-1215681-supp-final/Supplementary Material 1 Example of search strategy used in MedLine and adapted to search the other databases.pdf]

Supplementary Material 1: **Example of search strategy used in MedLine and adapted to search the other databases.**
• PICO physical exercise<br><br>MedLine, Embase, and Cochrane databases up to September 2019<br><br>((((((("Sedentary Behavior"[Mesh]) OR Sedentary Behavior) OR physical activity) OR "Exercise"[Mesh]) OR physical exercise) OR ((lifestyle) OR "Life Style"[Mesh]))) AND ((((((Lymphoma, Large B-Cell, Diffuse) OR "Lymphoma, Large B-Cell, Diffuse"[Mesh])) OR (((hodgkin lymphoma) OR "Hodgkin Disease"[Mesh]) OR hodgkin disease))) AND (("Cancer Survivors"[Mesh]) OR (cancer long term survivors))
• PICO nutrition and BMI<br><br>MedLine, Embase, and Cochrane databases up to September 2019<br><br>((((((((((("Hodgkin Disease"[Mesh]) OR hodgkin disease) OR hodgkin lymphoma)) OR (("Lymphoma, Large B-Cell, Diffuse"[Mesh]) OR Lymphoma, Large B-Cell, Diffuse))) AND ((cancer long term survivors) OR "Cancer Survivors"[Mesh])))))) AND ((((((Diet, Mediterranean[MeSH Terms]) OR diet[MeSH Major Topic]) OR "mediterranean diet") OR "Nutrition Therapy"[Mesh])) OR ((body mass index) OR body mass index[MeSH Terms]))
• PICO Dietary Supplements<br><br>MedLine, Embase, and Cochrane databases up to September 2019<br><br>(((((("Cancer Survivors"[Mesh]) OR cancer long term survivors)) AND (((((hodgkin lymphoma) OR hodgkin disease) OR "Hodgkin Disease"[Mesh]) OR lymphoma large B-cell diffuse) OR "Lymphoma, Large B-Cell, Diffuse"[Mesh]))) AND (((((((((dietary supplement) OR "Dietary Supplements"[Mesh]) OR essential fatty acids) OR "Fatty Acids, Essential"[Mesh]) OR vitamin) OR "Micronutrients"[Mesh]) OR "Amino Acids, Essential"[Mesh]) OR phytochemicals) OR "Phytochemicals"[Mesh] OR "natural supplements"))
• PICO cognitive decline<br><br>MedLine, Embase, and Cochrane databases up to October 2019<br><br>((((("hodgkin disease" OR hodgkin disease[mh] OR Lymphoma, Large B-Cell, Diffuse[MH] OR

DLBCL[TIAB] OR "diffuse large B cell lymphoma" OR "HODGKIN LYMPHOMA") AND ("surviv*" OR “long term survivor” OR “survivorship” OR cancer survivors[Mesh]))) AND ((((((Cognition Disorders[MH] OR Neurobehavioral Manifestations[mh] OR Attention[MH] OR Cognition[mh] OR Neuropsychological Tests[mh] OR Executive Function[MH] OR "executive functions"[tiab] OR ((cognit* OR neurocognit* OR neuropsycholog* OR memory OR neurobehavior* OR neurobehaviour*) AND (impair* OR deficit* OR declin* OR disorder* OR function* OR dysfunction* OR decrement* OR disturb* OR problem* OR sequelae* OR assess*)))))) OR ((("Anxiety"[Mesh] OR "Anxiety Disorders"[Mesh] OR anxi*[tiab] OR "Depression"[Mesh] OR depression[tiab] OR depressive[tiab] OR "Mood Disorders"[Mesh])))) OR ((sleep disorder) OR 'sleep disorder'[MeSH Terms]) OR (((((((("Exercise"[Mesh]) OR physical exercise) OR physical activity) OR "Sedentary Behavior"[Mesh] OR "Sedentary Behavior")))))
• PICO survivor cancer plan<br><br>MedLine, Embase, and Cochrane databases up to October 2019<br><br>((survivor cancer plan OR survivorship care plans)) AND (((("hodgkin disease" OR hodgkin disease[mh] OR Lymphoma, Large B-Cell, Diffuse[MH] OR DLBCL[TIAB] OR "diffuse large B cell lymphoma" OR "HODGKIN LYMPHOMA") AND ("surviv*" OR “long term survivor” OR “survivorship” OR cancer survivors[Mesh])))
